# Supplementary material for: Cryobanking European Mink (Mustela lutreola) Mesenchymal Stem Cells and Oocytes
Source: Int J Mol Sci. 2022 Aug 18;23(16):9319. doi: 10.3390/ijms23169319 (PMC9408899; doi:10.3390/ijms23169319)
Supplement: Supplementary file 1 [file ijms-23-09319-s001.zip › ijms-1830791-supplementary.pdf]

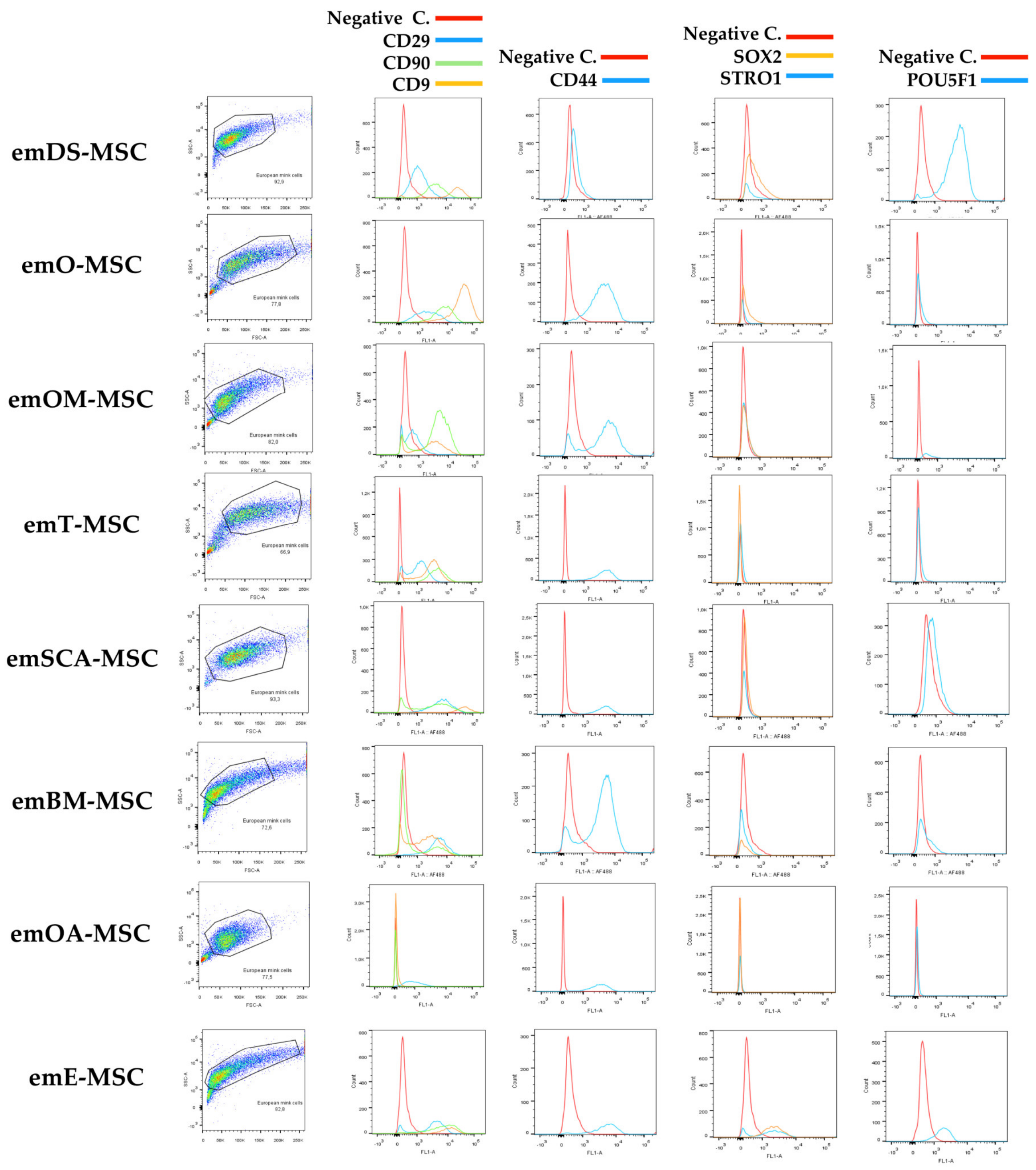

Figure S1. Analysis by flow cytometry of the expression levels of cell surface markers CD29, CD90, CD9, CD44, SOX2, STRO1, and POU5F1 in emDS-MSC, emO-MSC, emOM-MSC, emT-MSC, emSCA-MSC and emBM-MSC, emE-MSC and emOA-MSC. Plots depict the log of fluorescence intensity for each sample.
